# Supplementary material for: Long-term exposure to air pollution and severe COVID-19 in Catalonia: a population-based cohort study
Source: Nat Commun. 2023 May 24;14:2916. doi: 10.1038/s41467-023-38469-7 (PMC10209206; doi:10.1038/s41467-023-38469-7)
Supplement: Supplementary file 1 — Supplementary Information [file 41467_2023_38469_MOESM1_ESM.pdf]

Supplementary material

Table of Contents

Supplementary Methods 3

Catalonia Maps 3

Cohort construction 4

Exposure assessment 7

Analysis and model description 9

Missing data and multiple imputation 11

Supplementary Figure S1. Study Flowchart 12

Supplementary Table S1. Fully adjusted associations between long-term O_3 and COVID-19 related outcomes in single and two-pollutant models 13

Supplementary Figure S2. Sequential adjustment and sensitivity analyses for associations between long-term exposure to O_3 and COVID-19 related hospitalization (single pollutant models) 14

Supplementary Table S2. Unadjusted long-term associations between air pollutants and COVID-19 related outcomes in single and two-pollutant models 15

Supplementary Table S3. Fully adjusted long-term associations between air pollutants and COVID-19 related outcomes in single and two-pollutant models by 1 unit increase in air pollutants 16

Supplementary Figure S3. Sequential adjustment and sensitivity analyses for the association between long-term exposure to NO_2, $PM_{2.5}$, O_3 and COVID-19 related ICU admissions 17

Supplementary Figure S4. Sequential adjustment and sensitivity analyses for the association between long-term exposure to NO_2, $PM_{2.5}$, O_3 and COVID-19 related deaths 18

Supplementary Figure S5. Sequential adjustment and sensitivity analyses for the association between long-term exposure to NO_2, $PM_{2.5}$, O_3 and hospital length-of-stay .. 19

Supplementary table S4. Fully adjusted long-term associations between NO_2 (increase: 16.1) and COVID-19 related outcomes in single and two-pollutant models (additional sensitivity analyses) 20

Supplementary table S5. Fully adjusted long-term associations between $PM_{2.5}$ (increase: 3.2) and COVID-19 related outcomes in single and two-pollutant models (additional sensitivity analyses) 21

Supplementary table S6. Fully adjusted long-term associations between O_3 (increase: 10.8) and COVID-19 related outcomes in single and two-pollutant models (additional sensitivity analyses) 22

Supplementary Table S7. Adjusted long-term associations between O_3 and COVID-19 related outcomes in single-pollutant models by COVID-19 waves 23

Supplementary Table S8. Adjusted long-term associations between O3 and COVID-19 related hospitalization, in single and two-pollutant models, comparing all-cause with cause-specific hospitalizations 24

Supplementary Table S9. Causes of admission among the COVID-19 related hospitalization 25

Supplementary Table S10. Adjusted long-term associations between NO_2 and COVID-19 related outcomes, in single-pollutant models, comparing different cohorts (sensitivity analysis) 26

Supplementary Table S11. Adjusted long-term associations between $PM_{2.5}$ and COVID-19 related outcomes, in single-pollutant models, comparing different cohorts (sensitivity analysis) 27

Supplementary Table S12. Adjusted long-term associations between O_3 and COVID-19 related outcomes, in single-pollutant models, comparing different cohorts (sensitivity analysis) 28

Supplementary Table S13. Fully adjusted long-term associations between air pollutants and COVID-19 related events in single and two-pollutant models: COVAIR-CAT 2018 29

Supplementary Table S14. Fully adjusted long-term associations between air pollutants and COVID-19 related events in single and two-pollutant models: ELAPSE 2010 30

Supplementary Figure S6. Nonlinear exposure-response function between long-term exposure to NO_2 and $PM_{2.5}$ and COVID-19 related hospitalization in the main analysis 31

Supplementary Figure S7. Nonlinear exposure-response function between long-term exposure to NO_2 and $PM_{2.5}$ and COVID-19 related ICU admission in the main analysis 32

Supplementary Figure S8. Nonlinear exposure-response function between long-term exposure to NO_2 and $PM_{2.5}$ and COVID-19 related death in the main analysis 33

Supplementary table S15. Literature review on individual-level cohort studies and long-term exposure with severe COVID-19 outcomes 34

Supplementary Methods

Catalonia Maps

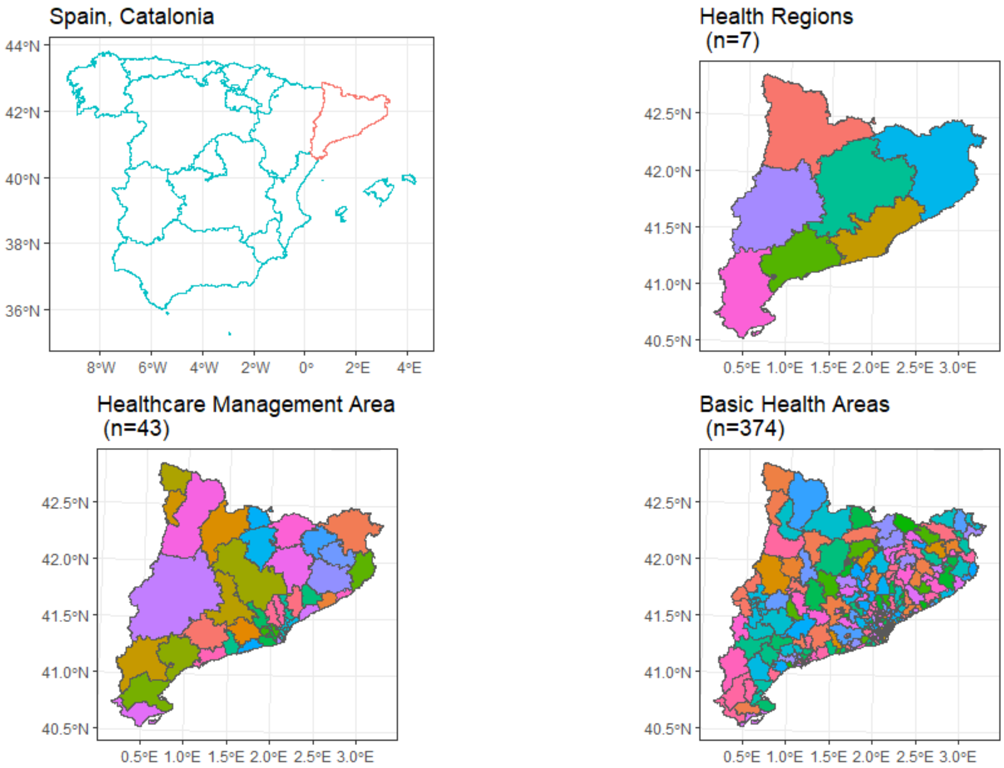

These maps were built with the software R (version 4.1.2) and using the public shapefiles (CC-BY 4.0) from

https://centrodedescargas.cnig.es/CentroDescargas/index.jsp
https://www.icgc.cat/ca/Descarregues/Cartografia-vectorial/Divisions-administratives
https://salutweb.gencat.cat/ca/el_departament/estadistiques_sanitaries/cartografia/

Cohort construction

Databases used

Name	Description
CMDB-AP	Primary care database
CMDB-HA	Hospital discharges database
CMDB-URG	Emergency care database
ECAP	Primary care source database
SUVEC	Epidemiological surveillance emergency service of Catalonia
RCA	Catalan Central Registry of Insured Individuals
AQuAS	Agency for Health Quality and Assessment of Catalonia

Variables definition

Variable	Content	Source	Defined by	Comments
Age	Age upon 01/01/2020, in years	RCA	Date of birth	Variable provided in categories of 3 years band because of confidentiality. We generated a random integer between the three possibilities to ascertain the final continuous age (e.g., for age category 50-52, we randomly select a number between 50, 51 and 52
Sex	Female / Male	RCA	Sex at birth	
Individual income group	Three categories of low (<18,000 thousand euros per year), middle (18,000-100,000 thousand euros) and high (>100,000 thousand euros)	RCA	Co-payment system for drug dispensations	This variable is already categorical. We considered individuals exempt from co-payment (nonworking population or people receiving non-contributory pension) as the low category.
Tobacco smoking status	Non-smoker / Former smoker / Active smoker	ECAP	General practitioner recording	For the main analysis, we considered those missing as non-smoker. This information was collected in 2015 and 2018; we used the latest available.
Health risk group (grupos de morbilidad	Healthy / Low / Moderate / High	RCA	Briefly, the index encompasses multimorbidity	To obtain the health status groups, we used the

Variable	Content	Source	Defined by	Comments
ajustados, GMA)			and levels of patient complexity, accounting for acute, chronic or oncological morbidities, if single or multi morbidity, medications and demand of the health system.	distribution of the score, as suggested, classifying those Health up to percentile 50th, Low up to 80th, Moderate up to 95th and High above 95th.[1] The index is associated with the use of healthcare resources.[2]
Diabetes mellitus	Yes / No	CMDB-AP and CMDB-HA	ICD-09: 250.x0, 250.x2, 357.2, 362.01, 362.02, 362.03, 362.04, 362.05, 362.06, 362.07, 366.41 249*, 250.x1, 250.x3<br>ICD-10: E11*, E12*, E13*	Diagnosis up to 01/03/2020
Obesity	Yes / No	CMDB-AP	ICD-10: E66*	Diagnosis up to 01/03/2020
Body-mass index	BMI, kg/m^2	ECAP	General practitioner recording	Information collected in 2015 and 2018; we used the latest available.
Chronic Obstructive Pulmonary Disorder	Yes / No	CMDB-AP and CMDB-HA	ICD-09: 491.0, 491.1, 491.8, 491.9, 491.20, 491.21, 491.22, 492.0, 492.8, 496*<br>ICD-10: J41*, J42*, J43*, *J44*	Diagnosis up to 01/03/2020
Hypertension	Yes / No	CMDB-AP	ICD-10: I10*	Diagnosis up to 01/03/2020
Other cardiovascular disorders	Yes / No	CMDB-AP and/or CMDB-HA	ICD-09: 410*, 433.x1, 434*<br>ICD-10: I10*, I48*, I20*, I21*, I22*, I23*, I24*, I25*, I61*, I63*, G45*,I65*, I66*, I67*, I67.2, I67.8, I67.9, I69*, I70*, I73, I73.9	Diagnosis up to 01/03/2020. It includes atrial fibrillation, acute myocardial infarction, stroke, other vascular disorders
Dyslipidemia	Yes / No	CMDB-AP	ICD-10: E78*	Diagnosis up to 01/03/2020
Nursing home status	Yes / No	SUVEC	Place of residence	Available for COVID-19 cases
Urbanicity (Area of residence)	City<br>Town and Suburb<br>Rural	RCA	1) Cities (Densely populated areas: at least 50 % of the population lives in urban centres) 2) Towns and suburbs (Intermediate density areas: less than 50 % of the population lives in rural grid cells and less than 50 % of the population lives in urban centres), 3) Rural	The boundaries of the degree of urbanisation areas are derived from the LAU2 2018 boundaries from the European Commission-Eurostat/GISO, based on data from EuroGeographics and the Eurostat 2011 Population grid

Variable	Content	Source	Defined by	Comments
			areas (Thinly populated areas: more than 50 % of the population lives in rural grid cells).	
Small Area Socioeconomic Index	Socioeconomic index at primary care service area level	RCA		Índex socioeconòmic territorial, based on data from 2017
Deprivation index	Deprivation index at primary care service area level	RCA/Census		Índice de privación 2011
Percentage of non-spanish residents	Proportion of non-spanish residents at census tract	RCA/Census		From 2018
Gini index	Inequity index at census tract	RCA/Census		Census 2011
Distance to closest primary care unit	Distance in meters from residence to closest primary care unit	RCA		2021
Average weekly TPP	Weekly test-positive proportion at AGA level	AQuaS	Aggregated public data	
Clinical COVID-19 diagnosis	Clinical diagnosis	CMDB-AP, CMDB-HA, CMDB-URG	ICD-10: B34.2, B97.2, J12.81, J12.89, U07.1	
Laboratory confirmed COVID-19 diagnosis	RT-PCR or Antigen test for SARS-CoV-2	SUVEC	Positive RT-PCR / Antigen test	
All-cause hospitalization after COVID-19 diagnosis	All-cause hospitalization after 30 days of first COVID-19 (clinical/ laboratory) diagnosis	CMDB-HA		We also allowed hospitalizations that occurred before 10 days of the diagnosis
Cardiovascular-related hospitalization after COVID-19 diagnosis	Cardiovascular cause as main reason of hospitalization after 30 days of first COVID-19 (clinical/ laboratory) diagnosis	CMDB-HA	ICD-10: I*	We also allowed hospitalizations that occurred before 10 days of the diagnosis
Respiratory-related hospitalization after COVID-19 diagnosis	Respiratory cause as main reason of hospitalization after 30 days of first COVID-19 (clinical/ laboratory) diagnosis	CMDB-HA	ICD-10: J*	We also allowed hospitalizations that occurred before 10 days of the diagnosis
Infection-related hospitalization after COVID-19 diagnosis	Infection cause as main reason of hospitalization after 30 days of first COVID-	CMDB-HA	ICD-10: A*	We also allowed hospitalizations that occurred before 10 days of

Variable	Content	Source	Defined by	Comments
	19 (clinical/ laboratory) diagnosis			the diagnosis
Ill defined-related hospitalization after COVID-19 diagnosis	Ill-defined cause as main reason of hospitalization after 30 days of first COVID-19 (clinical/ laboratory) diagnosis	CMDB-HA	ICD-10: R*	We also allowed hospitalizations that occurred before 10 days of the diagnosis
ICU admission after COVID-19 diagnosis	ICU admission during the all-cause hospitalization after 30 days of first COVID-19 (clinical/ laboratory) diagnosis	CMDB-HA		We also allowed hospitalizations that occurred before 10 days of the diagnosis
Hospital length of stay	Days of hospitalization during the all-cause hospitalization after 30 days of first COVDI-19 (clinical/laboratory) diagnosis	CMDB-HA		We also allowed hospitalizations that occurred before 10 days of the diagnosis

Exposure assessment

COVAIR-CAT (2019): Within the COVAIR-CAT project, we developed an exposure assessment for daily temperature, $PM_{2.5}$, PM_{10}, NO_2, and maximum 8h-average O_3 at a spatial resolution of 250m for the period 2018-2020 in Catalonia. We used meteorological and air pollution data from the Catalan and Spanish monitoring networks and applied machine learning methods tailored for spatio-temporal prediction (Random Forest (RF) -based spatial variable selection).[3,4] The list of predictors included meteorological (ERA5 and ERA5land reanalysis products) and atmospheric models (CAMS European reanalysis for year 2018 and analysis for 2019-2020, CAMS global reanalysis for Aerosol Optical Depth (AOD) and gas columns), remote sensing products (MODIS AOD and Land Surface Temperature (LST), OMI (Aura) tropospheric NO_2 and total O_3 columns, TROPOMI (Sentinel 5P) tropospheric NO_2 and total O_3 columns, VIIRS nighttime lights, Sentinel 2 NDVI), and a set of additional variables (road density, industrial emission point sources, land use indicators, terrain variables, tree cover and impervious surfaces density, coordinates, distance from sea, public holidays, dust advection days, julian day), as well as leave-on-out Inverse Distance Weighting estimates from the nearest stations to capture the spatial autocorrelation. The modelling framework was structured in two phases: First, we reconstructed the daily remote sensing products with missing pixels due to cloud cover (LST, AOD, OMI/TROPOMI gas columns) using temporally collocated atmospheric and climate models at the time of the satellite overpass, as well as other data such as land cover and terrain variables, using RF models. Secondly, we modelled the station data using the set of described predictors using a quantile RF model with uncertainty estimation. $PM_{2.5}$ models included a pre-processing step in which we predicted concentrations in stations with no records of $PM_{2.5}$ using PM_{10} data, which had a much larger monitoring network (35 vs. 91 stations). Assessment of the prediction

performance was done via nested 10-fold CV using the station as a grouping variable, i.e. out-of-station assessment.

Nested 10-fold CV performance statistics of COVAIR-CAT models (2019)

Exposure	RMSE	R2	bias	slope	$RMSE_{spatial}$	$R2_{spatial}$	$RMSE_{temporal}$	$R2_{temporal}$
$PM_{2.5}$ (ug/m3)	4.51	0.61	-1.17	1.08	2.07	0.71	4.05	0.56
NO_2 (ug/m3)	7.21	0.77	0.18	0.98	5.45	0.78	4.70	0.76
O_3 (ug/m3)	9.70	0.87	0.49	1.00	5.39	0.67	8.08	0.90

ELAPSE (2010): We used annual mean ambient exposure to $PM_{2.5}$, NO_2, O_3 and BC. In ELAPSE, standardized Europe-wide hybrid LUR models for 2010 were developed at 100 m spatial resolution.[5] We assigned to each study's participant the 2010 ELAPSE[6–8]-derived annual mean $PM_{2.5}$, NO_2, O_3 and BC estimates at each individual's residential address.

Description of the annual estimates for the air pollutants from COVAIR and ELAPSE models

Pollutant	Model	mean (SD) μ/m^3	Min-Max μ/m^3	median [p25-p75] μ/m^3	IQR μ/m^3
NO_2	COVAIR 2019	26.19 (10.3)	1.31-62.04	28.26 (17.98-34.06)	16.1
	ELAPSE 2010	35.45 (12.0)	1.17-87.21	35.84 (25.68-46.07)	20.4
$PM_{2.5}$	COVAIR 2019	13.85 (2.2)	5.18-21.06	13.9 (12.11-15.35)	3.2
	ELAPSE 2010	15.99 (1.8)	1.49-21.80	16.23 (14.87-17.32)	2.5
O_3	COVAIR 2019 (warm season)	91.64 (8.2)	61.35-113.97	92.47 (87.38-98.22)	10.8
	ELAPSE 2010 (average)	71.64 (7.2)	43.49-94.27	70.6 (65.76-77.33)	11.6
Black carbon	ELAPSE 2010	2.2 (0.4)	1.05-4.65	2.12 (1.85-2.57)	0.7

Spearman correlation coefficient of air pollutants between COVAIR-2019 and ELAPSE-2010 models

	**NO_2 (COVAIR)**	**$PM_{2.5}$ (COVAIR)**	**O_3 (warm season) (COVAIR)**	**NO_2 (ELAPSE)**	**$PM_{2.5}$ (ELAPSE)**	**O_3 (ELAPSE)**	**Black carbon (ELAPSE)**
**NO_2 (COVAIR)**	1						
**$PM_{2.5}$ (COVAIR)**	0.89	1					
**O_3 (warm season) (COVAIR)**	-0.82	-0.76	1				
**NO_2 (ELAPSE)**	0.88	0.83	-0.72	1			
**$PM_{2.5}$ (ELAPSE)**	0.78	0.76	-0.64	0.90	1		
**O_3 (ELAPSE)**	-0.84	-0.81	0.71	-0.96	-0.9	1	
**Black carbon (ELAPSE)**	0.83	0.76	-0.7	0.94	0.86	-0.93	1

Analysis and model description

**Model**	**Domain**	**Adjustment**
**Main analysis**		
Model 1 (M1)	Demographic	age (continuous term, penalized spline with 6 df) + sex (strata, 2 categories)
Model 2 (M2)	Demographic + Individual covariates	M1 + smoking status (factor, 3 categories) + individual income (factor, 3 categories) + health risk group (factor, 4 categories)
Model 3 (M3)	Demographic + Individual covariates + Area level covariates	M2 + Small Area Socioeconomic Index (continuous term) + Percentage of non-Spanish nationals (continuous term) + Distance to the closest Primary Care Unit (continuous term) + urbanicity (strata, 3 categories) + average weekly of test-positive proportion (continuous term)
Model 4 (M4) - Main Model	Demographic + Individual	M3 + Health region (strata, 7

Model	Domain	Adjustment
	covariates + Area level covariates + Health region indicator	categories)
**Sensitivity analyses**		
Model 5 (S1-M5)	Adding potential mediators	M4 (Main analysis) + Diabetes + COPD + Obesity + Dyslipidemia + Hypertension + Other cardiovascular disorders
Model 6 (S2-M6)	Additional socioeconomic indicators at area level	M4 (Main analysis) + Inequity index (Gini, continuous term) + Deprivation index (continuous term)
Model 7 (S3-M7)	After multiple imputation for BMI and Smoke status	M5 (S1-M5) + replacing smoke by imputed variable and replacing obesity by BMI (continuous term, penalized spline with 3 df)
Model 8 (S4-M8)	Analyzing only laboratory confirmed COVID-19	M4 (Population as main analysis but events accounted for laboratory confirmed COVID-19)
Model 9 (S5-M9)	Including COVID-19 cases diagnosed at nursing home	M4 (whole population including COVID-19 diagnosis at nursing home)
Model 10 (S6-M10)	Analyzing individuals that did not moved between	M4 (Population of not movers)
**Additional analyses**		
	Evaluating a time-stratified Cox PH model defining strata by Wave 1 and Wave 2	M4 (Main analysis) + exposure and strata(wave) interaction
	Hospitalizations defined by main cause of admission	M1, M2, M3, M4 (Main analysis), where outcome is defined when hospitalization occurred within 30 days of diagnosis and has COVID-19 or respiratory cause as main cause of admission
	Using COVAIR-2018 exposures	M4 (Main analysis) with exposure from 2018 from COVAIR instead of 2019
	Non-linearity of exposure-response function	M4 (Main analysis) testing single-pollutant models with penalized splines

Missing data and multiple imputation

We have missing data for two covariates: tobacco smoking and body-mass index. In the main analysis, only tobacco smoking is used as covariate, and we considered a missing value on tobacco smoking as non-smoker because the value is most often omitted for non-smokers in the primary care service. In a sensitivity analysis, we used multiple imputed smoking status. Body-mass index was used on a sensitivity analysis replacing obesity (yes/no) from the main analysis, after multiple imputation. Multiple imputation was conducted on 4,639,184 individuals after excluding COVID-19 cases diagnosed in nursing homes (main analysis population).

We conducted multiple imputation for covariates using multivariate chained equation methods using the mice package in R. After investigating the missingness pattern, we assumed a Missing at Random (MAR) mechanism. We used all covariates used in the full adjustment model (model 4), the exposure, outcome, and auxiliary variables (chronic comorbidities). Following the recommendations for imputation in time-to-event analysis, we also add the Nelson Aalen estimator in the model (White IR, 10.1002/sim.3618).

We generated 10 imputed datasets, with 10 iterations, and the estimates were pooled following the Rubin´s rule. We checked the variables distribution and convergence. Tobacco smoking was imputed using polytomous regression and body-mass index with linear regression methods. Below, a description of missing and imputed data.

Variable	Original data	Multiple imputed data
Tobacco smoking		
Never	1,861,340 (40.1%)	53.4% (53.4-53.5)
Former	678,411 (14.6%)	19.0% (19.0-19.1)
Current	944,152 (20.4%)	27.5% (27.4-27.5)
Missing*	1,155,281 (24.9%)	-
Body-mass index (kg/m^2)		
Mean ± SD	28.1 ± 5.73	27.4 ± 5.2
Median [p25-p75]	27.5 [24.1-31.2]	26.0 [24.4-30.1]
Missing	1,129,779	-

Supplementary Figure S1. **Study Flowchart**

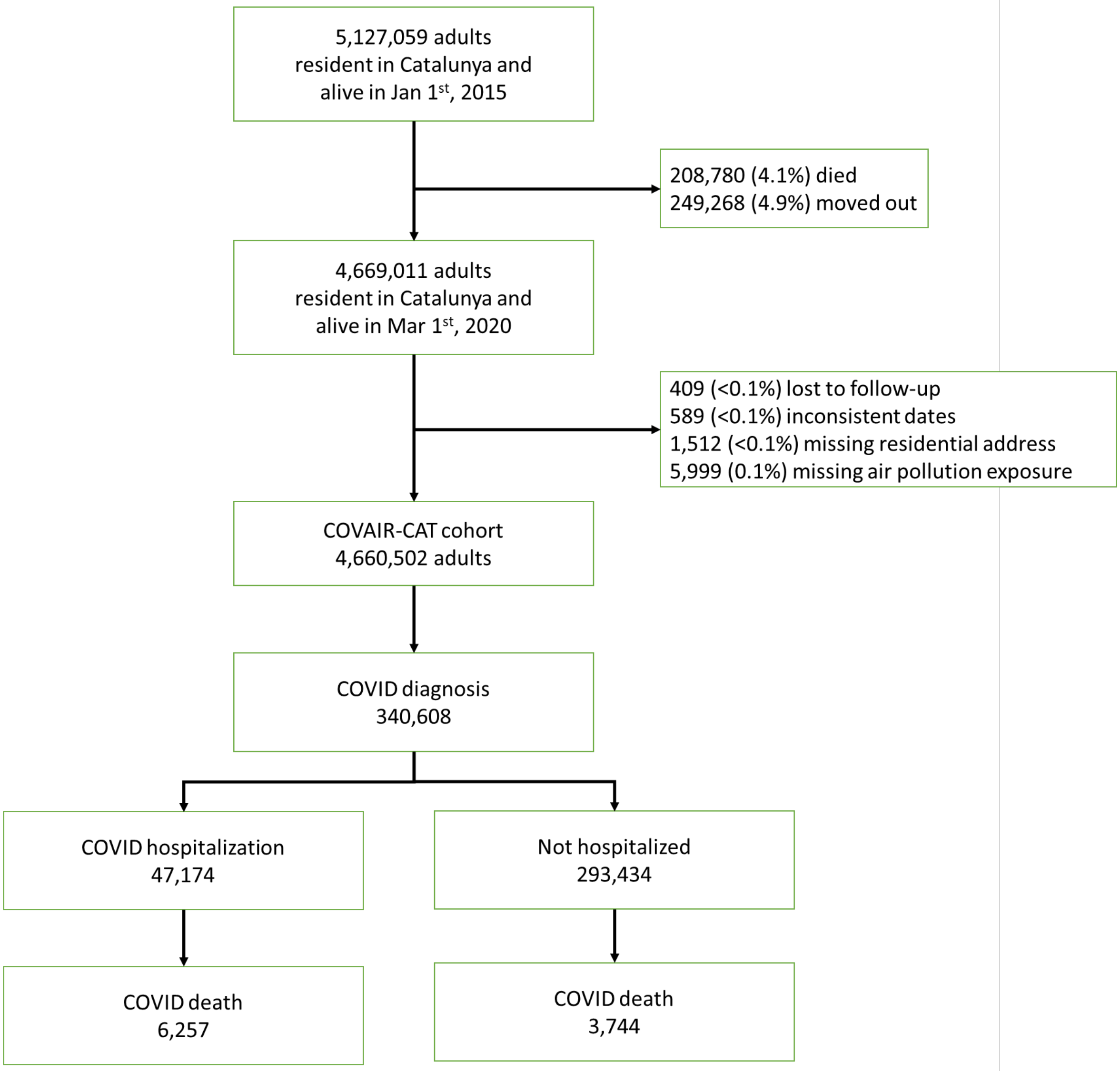

Supplementary Table S1. **Fully adjusted associations between long-term O_3 and COVID-19 related outcomes in single and two-pollutant models**

		**COVID-19 hospital admission**	**COVID-19 ICU admission**	**COVID-19 Death**	**Hospital length of stay**
	Exposure	HR (95% CI)	HR (95% CI)	HR (95% CI)	IRR (95% CI)
**O_3 (warm season)** (IQR increase: 10.8)	Single-pollutant	0.91 (0.89-0.92)	0.91 (0.86-0.96)	0.94 (0.90-0.98)	0.99 (0.97-1.00)
**O_3 (warm season)** (IQR increase: 10.8)	Adjust for NO_2	0.99 (0.97-1.01)	1.10 (1.02-1.18)	1.01 (0.95-1.07)	1.02 (0.99-1.04)
**O_3 (warm season)** (IQR increase: 10.8)	Adjusted for $PM_{2.5}$	0.97 (0.95-0.99)	0.96 (0.90-1.02)	0.98 (0.93-1.03)	1.01 (1.00-1.03)

Estimates from Model 4, which included: age (continuous term, penalized spline with 6 df) + sex (strata, 2 categories) + smoking status (factor, 3 categories) + individual income (factor, 3 categories) + health risk group (factor, 4 categories) + Small Area Socioeconomic Index (continuous term) + percentage of non-Spanish nationals (continuous term) + distance to the closest primary care unit (continuous term) + urbanicity (strata, 3 categories) + average weekly of test-positive proportion (continuous term) + health region (strata, 7 categories)

Supplementary Figure S2. **Sequential adjustment and sensitivity analyses for associations between long-term exposure to O_3 and COVID-19 related hospitalization** (single pollutant models)

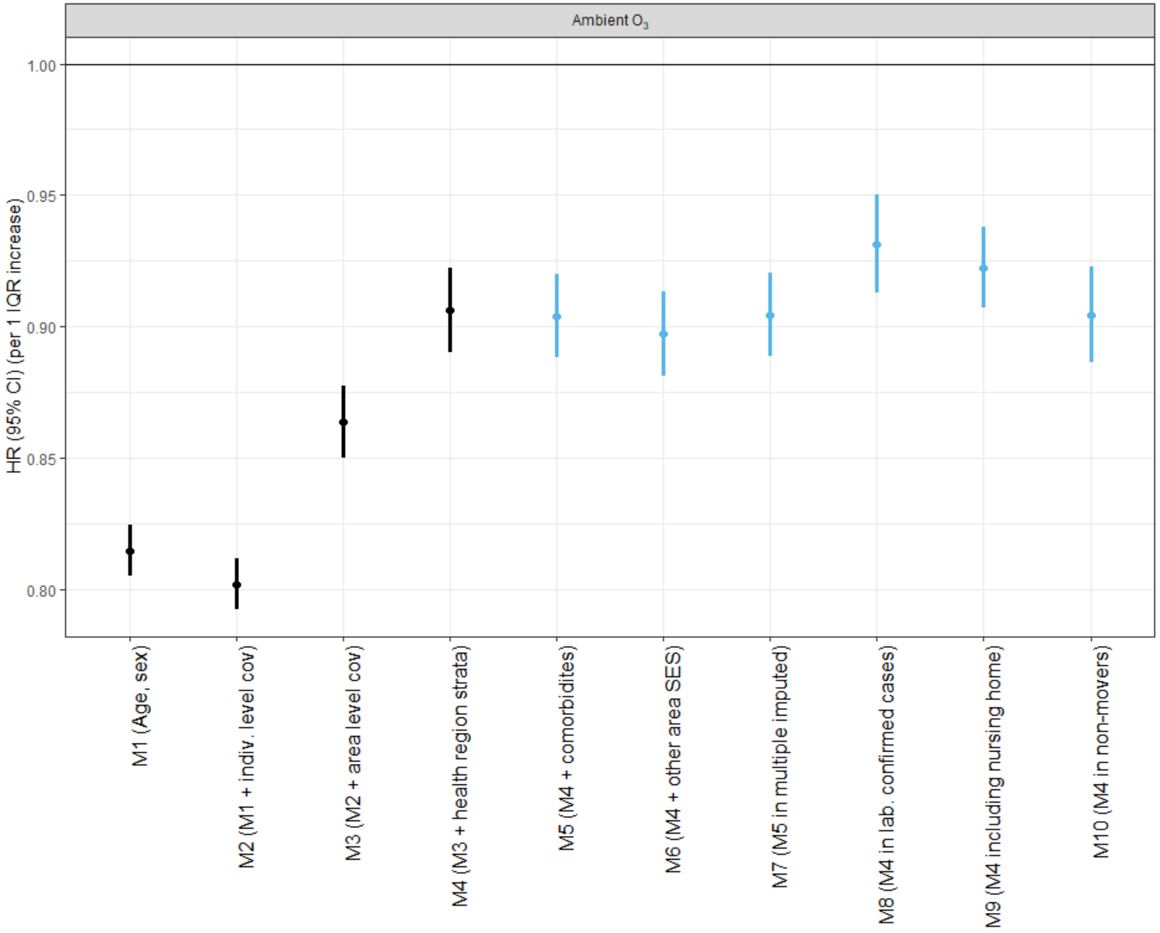

These estimates are from the sequential adjustment for confounding (black estimates, models 1-4) and six a priori sensitivity analyses (blue estimates, models 5-10), as described in the methods. Error bars refers to the 95% confidence interval from the Cox Proportional Hazards model. M denotes model; SES denotes socioeconomic status.

Supplementary Table S2. **Unadjusted long-term associations between air pollutants and COVID-19 related outcomes in single and two-pollutant models**

		**COVID-19 hospital admission**	**COVID-19 ICU admission**	**COVID-19 Death**	**Hospital length of stay**
	Exposure	HR (95% CI)	HR (95% CI)	HR (95% CI)	IRR (95% CI)
**NO_2** (IQR increase: 16.1)	Single-pollutant	1.45 (1.43-1.48)	1.45 (1.38-1.51)	1.43 (1.38-1.48)	1.05 (1.03-1.07)
**$PM_{2.5}$** (IQR increase: 3.2)	Single-pollutant	1.40 (1.38-1.42)	1.36 (1.31-1.42)	1.39 (1.34-1.43)	1.05 (1.03-1.06)
**O_3 (warm season)** (IQR increase: 10.8)	Single-pollutant	0.79 (0.78-0.80)	0.81 (0.78-0.84)	0.80 (0.78-0.82)	0.98 (0.97-0.99)
**NO_2** (IQR increase: 16.1)	Adjusted by PM2.5	1.24 (1.20-1.28)	1.34 (1.22-1.47)	1.19 (1.10-1.28)	1.02 (0.99-1.05)
**NO_2** (IQR increase: 16.1)	Adjust by O3	1.52 (1.48-1.56)	1.61 (1.48-1.75)	1.49 (1.40-1.59)	1.08 (1.05-1.11)
**$PM_{2.5}$** (IQR increase: 3.2)	Adjusted by NO2	1.18 (1.15-1.22)	1.08 (0.99-1.18)	1.21 (1.14-1.30)	1.04 (1.01-1.06)
**$PM_{2.5}$** (IQR increase: 3.2)	Adjust by O3	1.37 (1.34-1.39)	1.35 (1.27-1.43)	1.37 (1.31-1.44)	1.06 (1.04-1.08)
**O_3 (warm season)** (IQR increase: 10.8)	Adjusted by NO2	1.04 (1.02-1.07)	1.11 (1.04-1.18)	1.04 (0.99-1.10)	1.03 (1.01-1.05)
**O_3 (warm season)** (IQR increase: 10.8)	Adjust by PM25	0.97 (0.96-0.99)	0.99 (0.93-1.04)	0.99 (0.94-1.03)	1.02 (1.00-1.04)

Supplementary Table S3. **Fully adjusted long-term associations between air pollutants and COVID-19 related outcomes in single and two-pollutant models by 1 unit increase in air pollutants**

		**COVID-19 hospital admission**	**COVID-19 ICU admission**	**COVID-19 Death**	**Hospital length of stay**
	Exposure (1 unit increase)	HR (95% CI)	HR (95% CI)	HR (95% CI)	IRR (95% CI)
**NO_2**	Single-pollutant	1.014 (1.012-1.016)	1.022 (1.016-1.028)	1.010 (1.006-1.015)	1.003 (1.002-1.005)
**$PM_{2.5}$**	Single-pollutant	1.056 (1.049-1.063)	1.048 (1.026-1.070)	1.039 (1.022-1.056)	1.019 (1.013-1.026)
**O_3 (warm season)**	Single-pollutant	0.991 (0.989-0.993)	0.991 (0.986-0.996)	0.994 (0.990-0.998)	0.999 (0.997-1.000)
**NO_2**	Adjusted by PM2.5	1.007 (1.005-1.010)	1.026 (1.018-1.034)	1.006 (1.000-1.012)	0.999 (0.997-1.002)
	Adjust by O3	1.013 (1.011-1.016)	1.029 (1.021-1.037)	1.011 (1.005-1.017)	1.004 (1.002-1.007)
**$PM_{2.5}$**	Adjusted by NO2	1.036 (1.026-1.046)	0.979 (0.950-1.009)	1.023 (0.999-1.047)	1.021 (1.012-1.030)
	Adjust by O3	1.048 (1.040-1.057)	1.039 (1.014-1.064)	1.035 (1.016-1.055)	1.022 (1.015-1.030)
**O_3 (warm season)**	Adjusted by NO2	0.999 (0.997-1.001)	1.009 (1.002-1.016)	1.001 (0.995-1.006)	1.001 (0.999-1.003)
	Adjust by PM25	0.997 (0.995-0.999)	0.996 (0.990-1.002)	0.998 (0.994-1.003)	1.001 (1.000-1.003)

Model adjusted as Model 4: age (continuous term, penalized spline with 6 df) + sex (strata, 2 categories) + smoking status (factor, 3 categories) + individual income (factor, 3 categories) + health risk group (factor, 4 categories) + Small Area Socioeconomic Index (continuous term) + percentage of non-Spanish nationals (continuous term) + distance to the closest primary care unit (continuous term) + urbanicity (strata, 3 categories) + average weekly of test-positive proportion (continuous term) + health region (strata, 7 categories

Supplementary Figure S3. **Sequential adjustment and sensitivity analyses for the association between long-term exposure to NO_2, $PM_{2.5}$, O_3 and COVID-19 related ICU admissions**

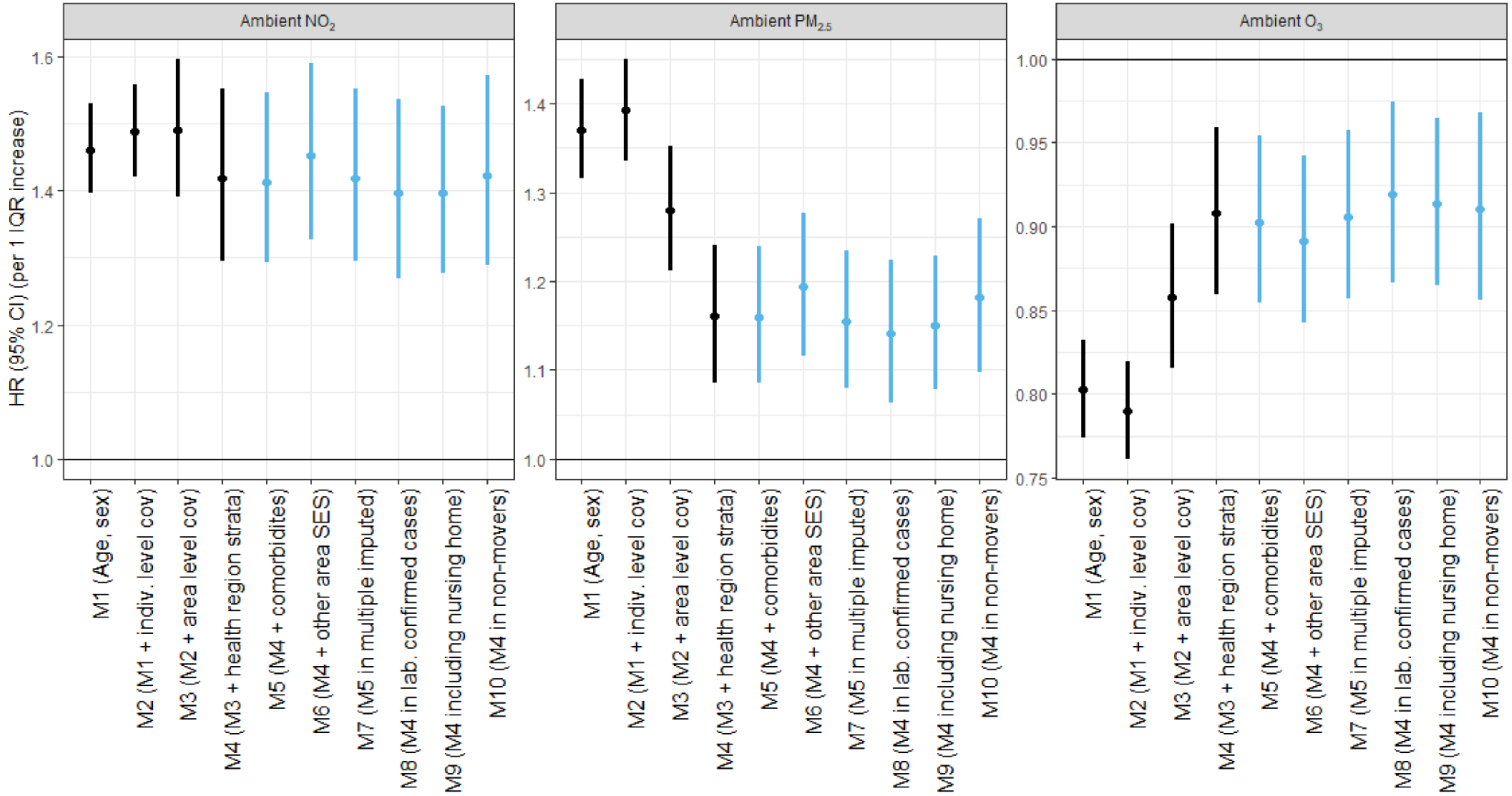

These estimates are from the sequential adjustment for confounding (black estimates, models 1-4) and six a priori sensitivity analyses (blue estimates, models 5-10), as described in the methods. Error bars refers to the 95% confidence interval from the Cox Proportional Hazards model. M denotes model; SES denotes socioeconomic status.

Supplementary Figure S4. **Sequential adjustment and sensitivity analyses for the association between long-term exposure to NO_2, $PM_{2.5}$, O_3 and COVID-19 related deaths**

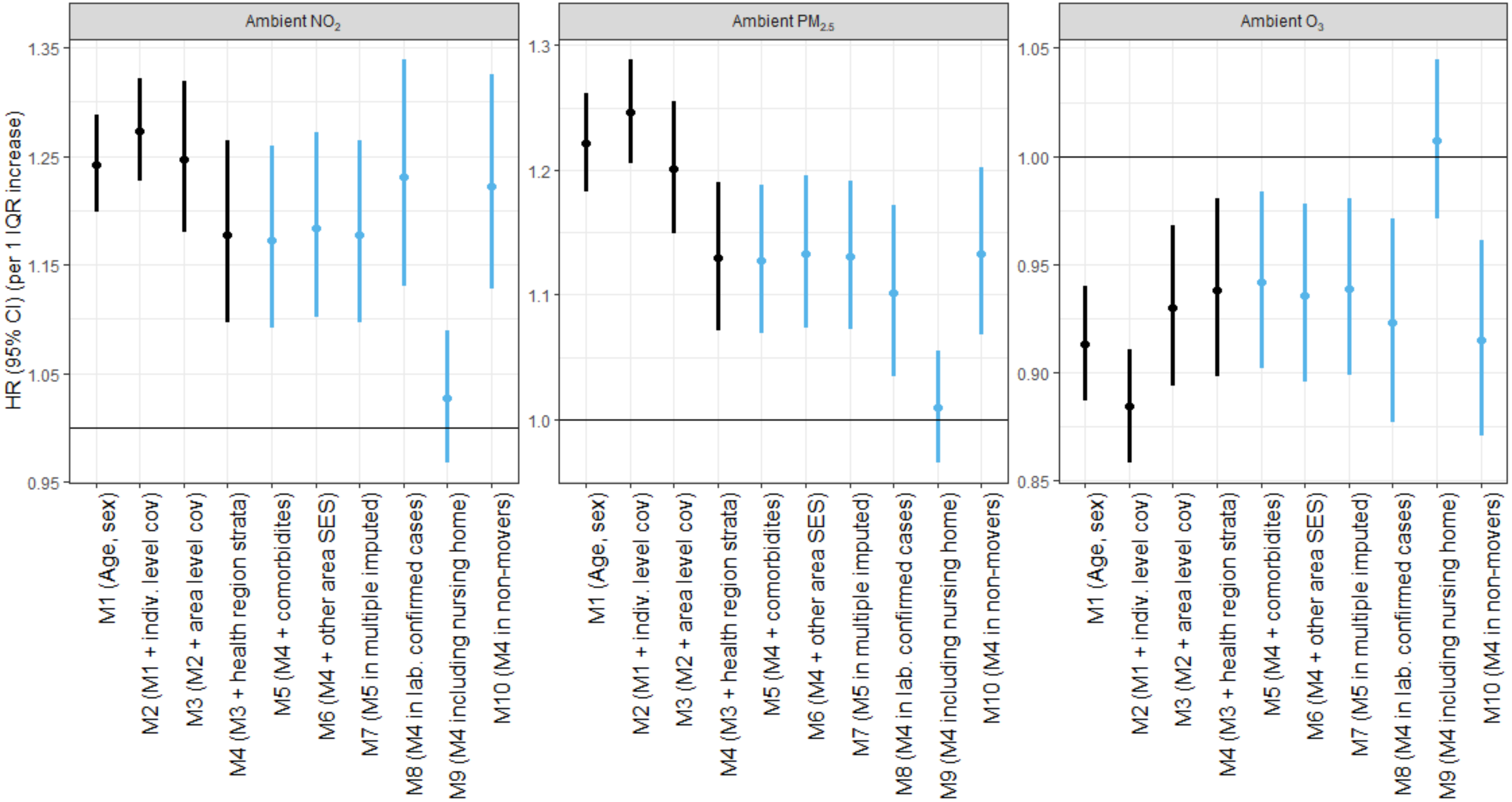

These estimates are from the sequential adjustment for confounding (black estimates, models 1-4) and six a priori sensitivity analyses (blue estimates, models 5-10), as described in the methods. Error bars refers to the 95% confidence interval from the Cox Proportional Hazards model. M denotes model; SES denotes socioeconomic status.

Supplementary Figure S5. **Sequential adjustment and sensitivity analyses for the association between long-term exposure to NO_2, $PM_{2.5}$, O_3 and hospital length-of-stay**

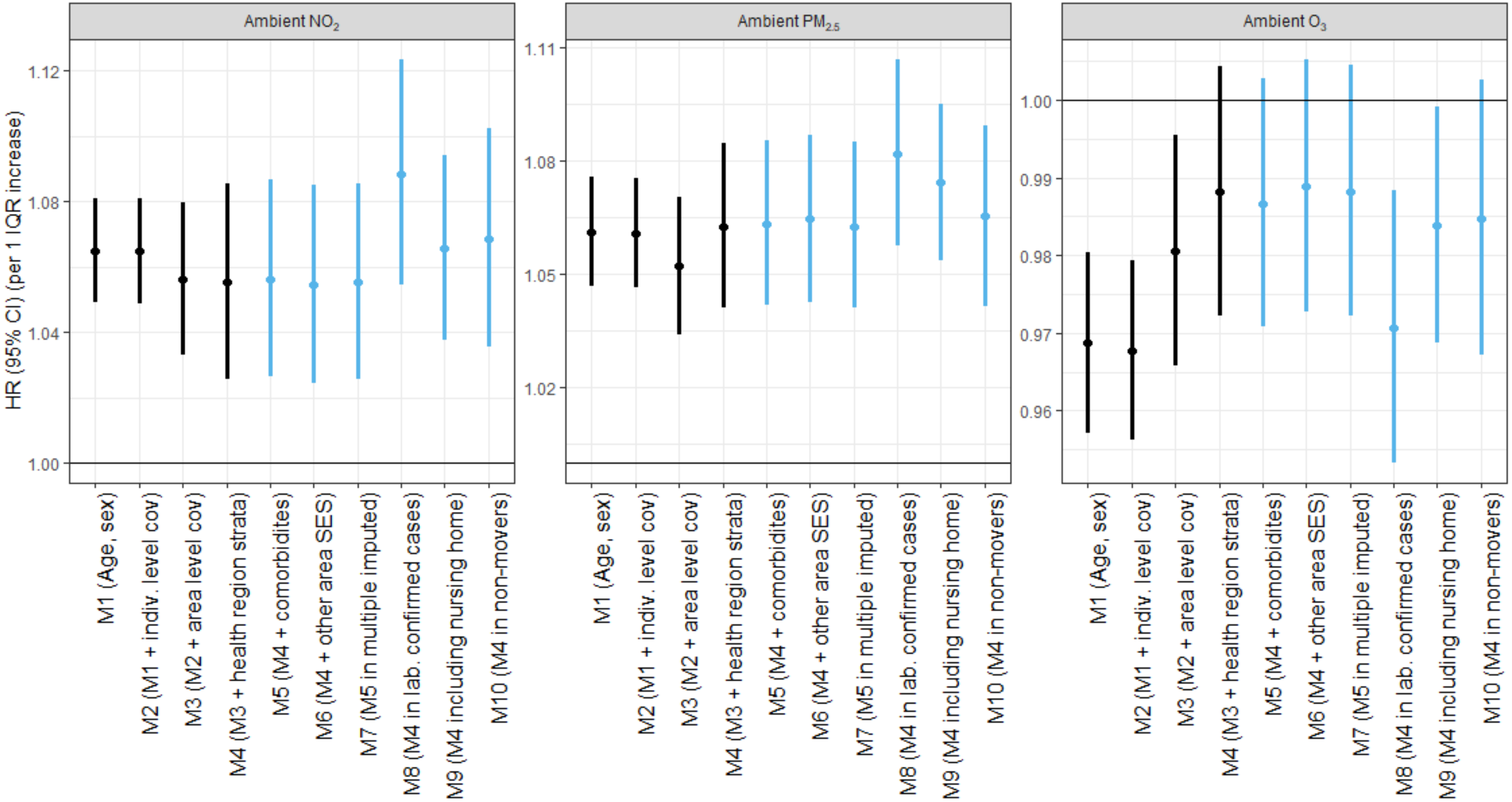

These estimates are from the sequential adjustment for confounding (black estimates, models 1-4) and six a priori sensitivity analyses (blue estimates, models 5-10), as described in the methods. Error bars refers to the 95% confidence interval from the Cox Proportional Hazards model. M denotes model; SES denotes socioeconomic status.

Supplementary table S4. **Fully adjusted long-term associations between NO_2 (increase: 16.1) and COVID-19 related outcomes in single and two-pollutant models (additional sensitivity analyses)**

	**COVID-19 hospital admission**	**COVID-19 ICU admission**	**COVID-19 Death**	**Hospital length of stay**
Analysis	HR (95% CI)	HR (95% CI)	HR (95% CI)	IRR (95% CI)
Main analysis (Model 4*)	1.25 (1.22-1.29)	1.42 (1.30-1.55)	1.18 (1.10-1.27)	1.06 (1.03-1.09)
Main analysis + follow-up until December 2020	1.25 (1.22-1.29)	1.42 (1.30-1.55)	1.18 (1.10-1.27)	1.06 (1.03-1.09)
Main analysis + adjusting for distance to the nearest hospital	1.22 (1.18-1.25)	1.40 (1.28-1.54)	1.16 (1.08-1.24)	1.06 (1.03-1.09)
Main analysis + adjusting for population density at the census tract level	1.23 (1.19-1.27)	1.37 (1.25-1.50)	1.19 (1.10-1.28)	1.06 (1.03-1.09)
Main analysis + adjusting for smoking using missing indicator	1.26 (1.22-1.29)	1.42 (1.30-1.56)	1.18 (1.10-1.26)	1.05 (1.03-1.09)

*Model 4: age (continuous term, penalized spline with 6 df) + sex (strata, 2 categories) + smoking status (factor, 3 categories) + individual income (factor, 3 categories) + health risk group (factor, 4 categories) + Small Area Socioeconomic Index (continuous term) + percentage of non-Spanish nationals (continuous term) + distance to the closest primary care unit (continuous term) + urbanicity (strata, 3 categories) + average weekly of test-positive proportion (continuous term) + health region (strata, 7 categories

Supplementary table S5. **Fully adjusted long-term associations between $PM_{2.5}$ (increase: 3.2) and COVID-19 related outcomes in single and two-pollutant models (additional sensitivity analyses)**

	**COVID-19 hospital admission**	**COVID-19 ICU admission**	**COVID-19 Death**	**Hospital length of stay**
Analysis	HR (95% CI)	HR (95% CI)	HR (95% CI)	IRR (95% CI)
Main analysis (Model 4*)	1.19 (1.16-1.21)	1.16 (1.09-1.24)	1.13 (1.07-1.19)	1.06 (1.04-1.08)
Main analysis + follow-up until December 2020	1.19 (1.16-1.21)	1.16 (1.09-1.24)	1.13 (1.07-1.19)	1.07 (1.04-1.09)
Main analysis + adjusting for distance to the nearest hospital	1.16 (1.14-1.19)	1.15 (1.07-1.23)	1.11 (1.06-1.18)	1.06 (1.04-1.09)
Main analysis + adjusting for population density at the census tract level	1.18 (1.15-1.20)	1.13 (1.06-1.21)	1.13 (1.08-1.20)	1.07 (1.04-1.09)
Main analysis + adjusting for smoking using missing indicator	1.18 (1.16-1.21)	1.15 (1.08-1.23)	1.13 (1.07-1.19)	1.06 (1.04-1.09)

*Model 4: age (continuous term, penalized spline with 6 df) + sex (strata, 2 categories) + smoking status (factor, 3 categories) + individual income (factor, 3 categories) + health risk group (factor, 4 categories) + Small Area Socioeconomic Index (continuous term) + percentage of non-Spanish nationals (continuous term) + distance to the closest primary care unit (continuous term) + urbanicity (strata, 3 categories) + average weekly of test-positive proportion (continuous term) + health region (strata, 7 categories

Supplementary table S6. **Fully adjusted long-term associations between O_3 (increase: 10.8) and COVID-19 related outcomes in single and two-pollutant models (additional sensitivity analyses)**

	**COVID-19 hospital admission**	**COVID-19 ICU admission**	**COVID-19 Death**	**Hospital length of stay**
Analysis	HR (95% CI)	HR (95% CI)	HR (95% CI)	IRR (95% CI)
Main analysis (Model 4*)	0.91 (0.89-0.92)	0.91 (0.86-0.96)	0.94 (0.90-0.98)	0.99 (0.97-1.00)
Main analysis + follow-up until December 2020	0.91 (0.89-0.92)	0.91 (0.86-0.96)	0.94 (0.90-0.98)	0.99 (0.97-1.01)
Main analysis + adjusting for distance to the nearest hospital	0.93 (0.91-0.94)	0.92 (0.87-0.97)	0.95 (0.91-1.00)	0.99 (0.97-1.00)
Main analysis + adjusting for population density at the census tract level	0.92 (0.90-0.94)	0.94 (0.88-0.99)	0.93 (0.89-0.98)	0.99 (0.97-1.00)
Main analysis + adjusting for smoking using missing indicator	0.90 (0.89-0.92)	0.90 (0.85-0.95)	0.94 (0.90-0.98)	0.99 (0.97-1.01)

*Model 4: age (continuous term, penalized spline with 6 df) + sex (strata, 2 categories) + smoking status (factor, 3 categories) + individual income (factor, 3 categories) + health risk group (factor, 4 categories) + Small Area Socioeconomic Index (continuous term) + percentage of non-Spanish nationals (continuous term) + distance to the closest primary care unit (continuous term) + urbanicity (strata, 3 categories) + average weekly of test-positive proportion (continuous term) + health region (strata, 7 categories

Supplementary Table S7. **Adjusted long-term associations between O_3 and COVID-19 related outcomes in single-pollutant models by COVID-19 waves**

		**First wave**	**Second wave**
	Exposure	HR (95% CI)	HR (95% CI)
**Hospitalization**			
	**O_3 (warm season)** (IQR increase: 10.8)	0.88 (0.86-0.90)	0.95 (0.92-0.97)
**ICU admission**			
	**O_3 (warm season)** (IQR increase: 10.8)	0.90 (0.84-0.97)	0.92 (0.85-0.99)
**Death**			
	**O_3 (warm season)** (IQR increase: 10.8)	0.94 (0.90-0.99)	0.93 (0.86-1.00)
**Hospital LOS**			
	**O_3 (warm season)** (IQR increase: 10.8)	0.99 (0.97-1.01)	0.99 (0.97-1.02)

Time-stratified Cox model adjusted as Model 4: age (continuous term, penalized spline with 6 df) + sex (strata, 2 categories) + smoking status (factor, 3 categories) + individual income (factor, 3 categories) + health risk group (factor, 4 categories) + Small Area Socioeconomic Index (continuous term) + percentage of non-Spanish nationals (continuous term) + distance to the closest primary care unit (continuous term) + urbanicity (strata, 3 categories) + average weekly of test-positive proportion (continuous term) + health region (strata, 7 categories)

Supplementary Table S8. **Adjusted long-term associations between O3 and COVID-19 related hospitalization, in single and two-pollutant models, comparing all-cause with cause-specific hospitalizations**

		**All cause (n=47,174)**	**COVID-19 or Respiratory* (n=36,505)**	**COVID-19* (n=33,981)**
	Exposure	HR (95% CI)	HR (95% CI)	HR (95% CI)
COVAIR models				
**O_3 (warm season)** (increase: 10.8)	Single-pollutant	0.91 (0.89-0.92)	0.91 (0.89-0.93)	0.90 (0.88-0.92)
COVAIR models				
**O_3 (warm season)** (increase: 10.8)	Adjusted for PM2.5	0.97 (0.95-0.99)	0.98 (0.95-1.00)	0.96 (0.94-0.99)
**O_3 (warm season)** (increase: 10.8)	Adjusted for NO2	0.99 (0.97-1.01)	1.01 (0.98-1.04)	0.99 (0.96-1.02)

* Defined by the ICD-10 code first position.

Model adjusted as Model 4: age (continuous term, penalized spline with 6 df) + sex (strata, 2 categories) + smoking status (factor, 3 categories) + individual income (factor, 3 categories) + health risk group (factor, 4 categories) + Small Area Socioeconomic Index (continuous term) + percentage of non-Spanish nationals (continuous term) + distance to the closest primary care unit (continuous term) + urbanicity (strata, 3 categories) + average weekly of test-positive proportion (continuous term) + health region (strata, 7 categories)

Supplementary Table S9. **Causes of admission among the COVID-19 related hospitalization**

**Cause**	**N**
All-cause hospital admission	47,174
Any mention to COVID-19	37,942 (80.4%)
COVID-19 as main cause of admission	33,981 (72.0%)
COVID-19 as secondary cause of admission	3,961 (8.4%)
Respiratory	2,524 (5.4%)
Cardiovascular	613 (1.3%)
Ill-defined	526 (1.1%)
Infection	129 (0.3%)
Other	5,440 (11.5%)

Causes defined by ICD-10 codes based on the COVID-19 codes and ICD chapters.

Supplementary Table S10. **Adjusted long-term associations between NO_2 and COVID-19 related outcomes, in single-pollutant models, comparing different cohorts (sensitivity analysis)**

		**Whole period**	**First wave**	**Second wave**
	Analysis	HR (95% CI)	HR (95% CI)	HR (95% CI)
Hospital admission				
**NO_2** (increase: 16.1)	Main analysis	1.25 (1.22-1.29)	1.32 (1.27-1.37)	1.16 (1.11-1.22)
**NO_2** (increase: 16.1)	Cases only	1.11 (1.08-1.15)	1.11 (1.07-1.15)	1.07 (1.02-1.12)
**NO_2** (increase: 16.1)	Primary care cases only	1.17 (1.13-1.22)	1.16 (1.11-1.21)	1.12 (1.05-1.19)
ICU admission				
**NO_2** (increase: 16.1)	Main analysis	1.42 (1.30-1.55)	1.48 (1.32-1.67)	1.34 (1.18-1.53)
**NO_2** (increase: 16.1)	Cases only	1.16 (1.05-1.27)	1.16 (1.03-1.31)	1.18 (1.02-1.35)
**NO_2** (increase: 16.1)	Primary care cases only	1.16 (1.02-1.32)	1.15 (0.98-1.35)	1.22 (1.01-1.48)
Death				
**NO_2** (increase: 16.1)	Main analysis	1.18 (1.10-1.27)	1.15 (1.06-1.25)	1.25 (1.10-1.41)
**NO_2** (increase: 16.1)	Cases only	1.00 (0.93-1.08)	0.92 (0.84-1.00)	1.13 (1.00-1.28)
**NO_2** (increase: 16.1)	Primary care cases only	1.00 (0.90-1.12)	0.87 (0.77-0.99)	1.33 (1.07-1.65)

*Model 4: age (continuous term, penalized spline with 6 df) + sex (strata, 2 categories) + smoking status (factor, 3 categories) + individual income (factor, 3 categories) + health risk group (factor, 4 categories) + Small Area Socioeconomic Index (continuous term) + percentage of non-Spanish nationals (continuous term) + distance to the closest primary care unit (continuous term) + urbanicity (strata, 3 categories) + average weekly of test-positive proportion (continuous term) + health region (strata, 7 categories

Supplementary Table S11. **Adjusted long-term associations between $PM_{2.5}$ and COVID-19 related outcomes, in single-pollutant models, comparing different cohorts (sensitivity analysis)**

		**Whole period**	**First wave**	**Second wave**
	Analysis	HR (95% CI)	HR (95% CI)	HR (95% CI)
Hospital admission				
**$PM_{2.5}$** (IQR increase: 3.2)	Main analysis	1.19 (1.16-1.21)	1.25 (1.21-1.28)	1.11 (1.07-1.14)
**$PM_{2.5}$** (IQR increase: 3.2)	Cases only	1.11 (1.08-1.13)	1.07 (1.04-1.10)	1.09 (1.05-1.12)
**$PM_{2.5}$** (IQR increase: 3.2)	Primary care cases only	1.15 (1.11-1.18)	1.12 (1.08-1.16)	1.11 (1.06-1.16)
ICU admission				
**$PM_{2.5}$** (IQR increase: 3.2)	Main analysis	1.16 (1.09-1.24)	1.19 (1.09-1.30)	1.12 (1.02-1.23)
**$PM_{2.5}$** (IQR increase: 3.2)	Cases only	0.98 (0.92-1.05)	0.97 (0.89-1.06)	1.02 (0.92-1.13)
**$PM_{2.5}$** (IQR increase: 3.2)	Primary care cases only	0.94 (0.85-1.03)	0.91 (0.81-1.03)	1.02 (0.89-1.18)
Death				
**$PM_{2.5}$** (IQR increase: 3.2)	Main analysis	1.13 (1.07-1.19)	1.12 (1.06-1.20)	1.14 (1.04-1.25)
**$PM_{2.5}$** (IQR increase: 3.2)	Cases only	1.00 (0.94-1.05)	0.91 (0.86-0.97)	1.08 (0.99-1.19)
**$PM_{2.5}$** (IQR increase: 3.2)	Primary care cases only	1.00 (0.92-1.09)	0.90 (0.82-0.99)	1.12 (0.94-1.32)

*Model 4: age (continuous term, penalized spline with 6 df) + sex (strata, 2 categories) + smoking status (factor, 3 categories) + individual income (factor, 3 categories) + health risk group (factor, 4 categories) + Small Area Socioeconomic Index (continuous term) + percentage of non-Spanish nationals (continuous term) + distance to the closest primary care unit (continuous term) + urbanicity (strata, 3 categories) + average weekly of test-positive proportion (continuous term) + health region (strata, 7 categories

Supplementary Table S12. **Adjusted long-term associations between O_3 and COVID-19 related outcomes, in single-pollutant models, comparing different cohorts (sensitivity analysis)**

		**Whole period**	**First wave**	**Second wave**
	Analysis	HR (95% CI)	HR (95% CI)	HR (95% CI)
Hospital admission				
**O_3 (warm season)** (IQR increase: 10.8)	Main analysis	0.91 (0.89-0.92)	0.88 (0.86-0.90)	0.95 (0.92-0.97)
**O_3 (warm season)** (IQR increase: 10.8)	Cases only	0.96 (0.94-0.98)	0.98 (0.96-1.00)	0.97 (0.94-1.00)
**O_3 (warm season)** (IQR increase: 10.8)	Primary care cases only	0.94 (0.91-0.96)	0.96 (0.93-0.99)	0.94 (0.91-0.98)
ICU admission				
**O_3 (warm season)** (IQR increase: 10.8)	Main analysis	0.91 (0.86-0.96)	0.90 (0.84-0.97)	0.92 (0.85-0.99)
**O_3 (warm season)** (IQR increase: 10.8)	Cases only	0.99 (0.94-1.05)	1.00 (0.93-1.07)	0.97 (0.89-1.05)
**O_3 (warm season)** (IQR increase: 10.8)	Primary care cases only	1.01 (0.94-1.09)	1.03 (0.94-1.14)	0.96 (0.86-1.07)
Death				
**O_3 (warm season)** (IQR increase: 10.8)	Main analysis	0.94 (0.90-0.98)	0.94 (0.90-0.99)	0.93 (0.86-1.00)
**O_3 (warm season)** (IQR increase: 10.8)	Cases only	1.02 (0.98-1.07)	1.09 (1.03-1.14)	0.96 (0.89-1.04)
**O_3 (warm season)** (IQR increase: 10.8)	Primary care cases only	1.06 (0.99-1.14)	1.15 (1.07-1.24)	0.96 (0.84-1.09)

*Model 4: age (continuous term, penalized spline with 6 df) + sex (strata, 2 categories) + smoking status (factor, 3 categories) + individual income (factor, 3 categories) + health risk group (factor, 4 categories) + Small Area Socioeconomic Index (continuous term) + percentage of non-Spanish nationals (continuous term) + distance to the closest primary care unit (continuous term) + urbanicity (strata, 3 categories) + average weekly of test-positive proportion (continuous term) + health region (strata, 7 categories

Supplementary Table S13. **Fully adjusted long-term associations between air pollutants and COVID-19 related events in single and two-pollutant models: COVAIR-CAT 2018**

		COVID-19 hospital admission	COVID-19 ICU admission	COVID-19 Death	Hospital length of stay
	Exposure	HR (95% CI)	HR (95% CI)	HR (95% CI)	IRR (95% CI)
**NO_2** (increase: 16.4)	Single-pollutant	1.22 (1.19-1.25)	1.32 (1.21-1.43)	1.15 (1.08-1.23)	1.06 (1.04-1.09)
**$PM_{2.5}$** (increase: 2.6)	Single-pollutant	1.17 (1.14-1.20)	1.16 (1.08-1.25)	1.13 (1.07-1.20)	1.06 (1.04-1.09)
**O_3 (warm)** (increase: 10.3)	Single-pollutant	0.89 (0.88-0.91)	0.90 (0.86-0.95)	0.92 (0.88-0.96)	0.98 (0.96-0.99)
**NO_2** (increase: 16.4)	Adjusted by PM2.5	1.15 (1.10-1.19)	1.36 (1.21-1.53)	1.08 (0.98-1.19)	1.01 (0.98-1.05)
	Adjusted for O3	1.17 (1.12-1.22)	1.45 (1.27-1.65)	1.10 (1.00-1.22)	1.07 (1.03-1.12)
**$PM_{2.5}$** (increase: 2.6)	Adjusted for NO2	1.08 (1.04-1.11)	0.96 (0.87-1.07)	1.08 (1.00-1.17)	1.05 (1.02-1.09)
	Adjusted for O3	1.11 (1.08-1.14)	1.12 (1.02-1.22)	1.09 (1.02-1.17)	1.06 (1.04-1.09)
**O_3 (warm)** (increase: 10.3)	Adjusted for PM25	0.94 (0.92-0.96)	0.95 (0.89-1.01)	0.95 (0.91-1.00)	1.00 (0.98-1.02)
	Adjusted for NO2	0.96 (0.94-0.99)	1.08 (1.00-1.17)	0.96 (0.90-1.03)	1.01 (0.99-1.03)

Model adjusted as Model 4: age (continuous term, penalized spline with 6 df) + sex (strata, 2 categories) + smoking status (factor, 3 categories) + individual income (factor, 3 categories) + health risk group (factor, 4 categories) + Small Area Socioeconomic Index (continuous term) + percentage of non-Spanish nationals (continuous term) + distance to the closest primary care unit (continuous term) + urbanicity (strata, 3 categories) + average weekly of test-positive proportion (continuous term) + health region (strata, 7 categories)

Supplementary Table S14. **Fully adjusted long-term associations between air pollutants and COVID-19 related events in single and two-pollutant models: ELAPSE 2010**

		COVID-19 hospital admission	COVID-19 ICU admission	COVID-19 Death	Hospital length of stay
	Exposure	HR (95% CI)	HR (95% CI)	HR (95% CI)	IRR (95% CI)
**NO_2** (increase: 20.4)	Single-pollutant	1.33 (1.29-1.38)	1.34 (1.21-1.49)	1.18 (1.08-1.28)	1.11 (1.08-1.15)
**$PM_{2.5}$** (increase: 2.5)	Single-pollutant	1.16 (1.13-1.18)	1.24 (1.16-1.33)	1.11 (1.05-1.17)	1.06 (1.04-1.08)
**O_3 (annual)** (increase: 11.6)	Single-pollutant	0.74 (0.72-0.76)	0.73 (0.66-0.81)	0.78 (0.72-0.85)	0.97 (0.94-1.00)
**Black carbon** (increase: 0.7)	Single-pollutant	1.19 (1.16-1.22)	1.19 (1.10-1.28)	1.06 (1.00-1.13)	1.04 (1.02-1.07)
**NO_2** (increase: 20.4)	Adjusted for PM2.5	1.31 (1.24-1.39)	1.07 (0.90-1.27)	1.09 (0.95-1.26)	1.09 (1.04-1.15)
	Adjusted for O3	1.01 (0.95-1.09)	1.02 (0.82-1.26)	0.77 (0.65-0.92)	1.34 (1.26-1.43)
	Adjusted for BC	1.34 (1.25-1.43)	1.40 (1.15-1.72)	1.38 (1.17-1.62)	1.23 (1.16-1.31)
**$PM_{2.5}$** (increase: 2.5)	Adjusted for NO2	1.01 (0.98-1.05)	1.21 (1.08-1.34)	1.06 (0.97-1.16)	1.01 (0.98-1.05)
	Adjusted for O3	0.98 (0.94-1.01)	1.14 (1.03-1.27)	0.94 (0.87-1.03)	1.10 (1.06-1.13)
	Adjusted for BC	1.08 (1.05-1.12)	1.24 (1.13-1.36)	1.13 (1.05-1.22)	1.06 (1.03-1.09)
**O_3 (annual)** (increase: 11.6)	Adjusted for PM25	0.72 (0.69-0.76)	0.85 (0.73-0.99)	0.74 (0.65-0.83)	1.08 (1.03-1.13)
	Adjusted for NO2	0.75 (0.70-0.80)	0.74 (0.61-0.90)	0.64 (0.55-0.75)	1.22 (1.15-1.30)
	Adjust by BC	0.70 (0.66-0.74)	0.67 (0.57-0.80)	0.59 (0.51-0.68)	1.03 (0.98-1.09)
**Black carbon** (increase: 0.7)	Adjusted for PM25	1.12 (1.08-1.16)	1.01 (0.91-1.12)	0.97 (0.89-1.05)	0.99 (0.96-1.03)
	Adjusted for NO2	1.00 (0.95-1.05)	0.96 (0.83-1.11)	0.88 (0.78-0.98)	0.92 (0.87-0.96)
	Adjusted for O3	0.95 (0.91-0.99)	0.92 (0.80-1.05)	0.76 (0.68-0.84)	1.06 (1.02-1.11)

Model adjusted as Model 4: age (continuous term, penalized spline with 6 df) + sex (strata, 2 categories) + smoking status (factor, 3 categories) + individual income (factor, 3 categories) + health risk group (factor, 4 categories) + Small Area Socioeconomic Index (continuous term) + percentage of non-Spanish nationals (continuous term) + distance to the closest primary care unit (continuous term) + urbanicity (strata, 3 categories) + average weekly of test-positive proportion (continuous term) + health region (strata, 7 categories

Supplementary Figure S6. **Nonlinear exposure-response function between long-term exposure to NO_2 and $PM_{2.5}$ and COVID-19 related hospitalization in the main analysis**

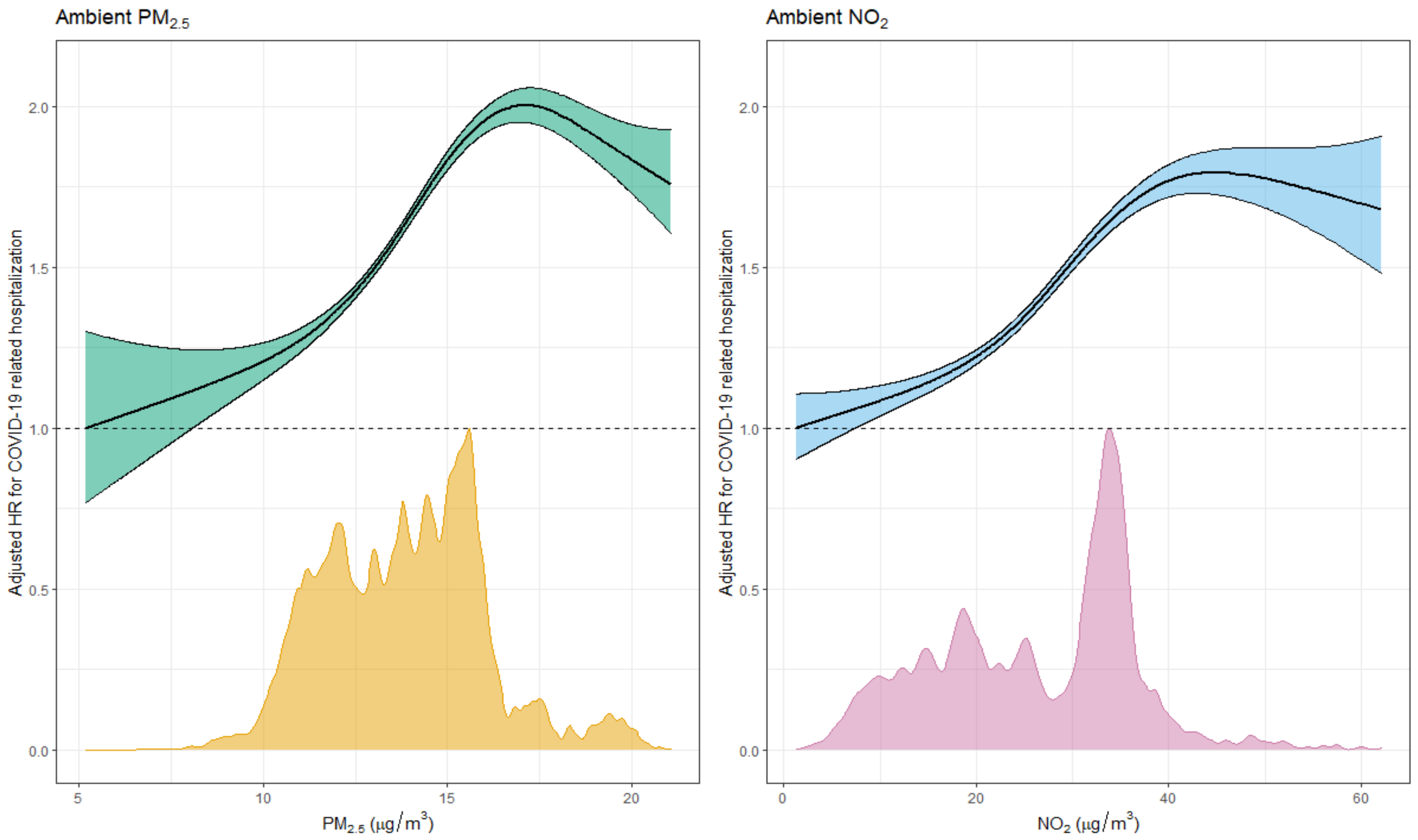

Model adjusted as Model 4: age (continuous term, penalized spline with 6 df) + sex (strata, 2 categories) + smoking status (factor, 3 categories) + individual income (factor, 3 categories) + health risk group (factor, 4 categories) + Small Area Socioeconomic Index (continuous term) + percentage of non-Spanish nationals (continuous term) + distance to the closest primary care unit (continuous term) + urbanicity (strata, 3 categories) + average weekly of test-positive proportion (continuous term) + health region (strata, 7 categories). Error bar bands refer to the 95% confidence interval from the Cox Proportional Hazards model.

Supplementary Figure S7. **Nonlinear exposure-response function between long-term exposure to NO_2 and $PM_{2.5}$ and COVID-19 related ICU admission in the main analysis**

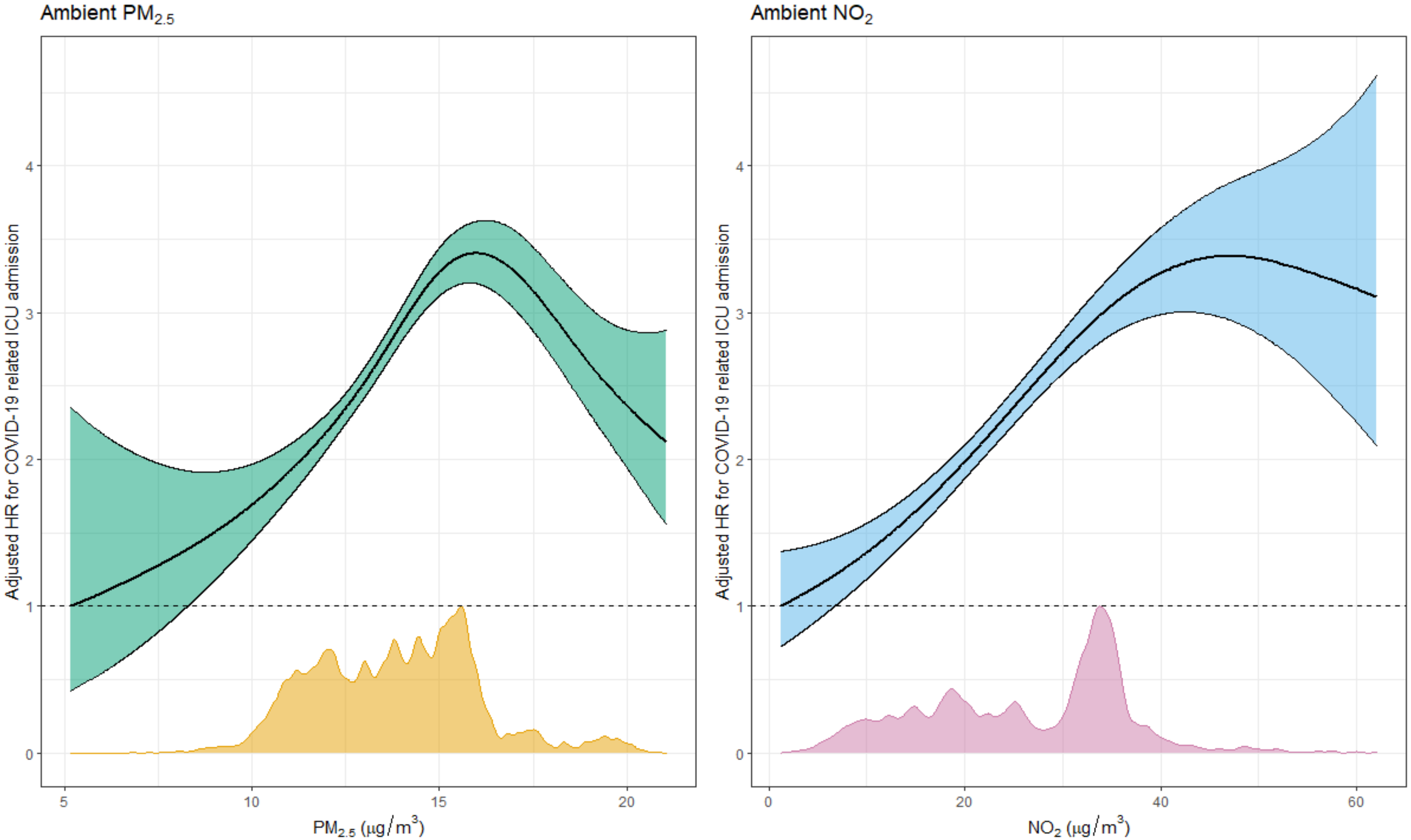

Model adjusted as Model 4: age (continuous term, penalized spline with 6 df) + sex (strata, 2 categories) + smoking status (factor, 3 categories) + individual income (factor, 3 categories) + health risk group (factor, 4 categories) + Small Area Socioeconomic Index (continuous term) + percentage of non-Spanish nationals (continuous term) + distance to the closest primary care unit (continuous term) + urbanicity (strata, 3 categories) + average weekly of test-positive proportion (continuous term) + health region (strata, 7 categories). Error bar bands refer to the 95% confidence interval from the Cox Proportional Hazards model.

Supplementary Figure S8. **Nonlinear exposure-response function between long-term exposure to NO_2 and $PM_{2.5}$ and COVID-19 related death in the main analysis**

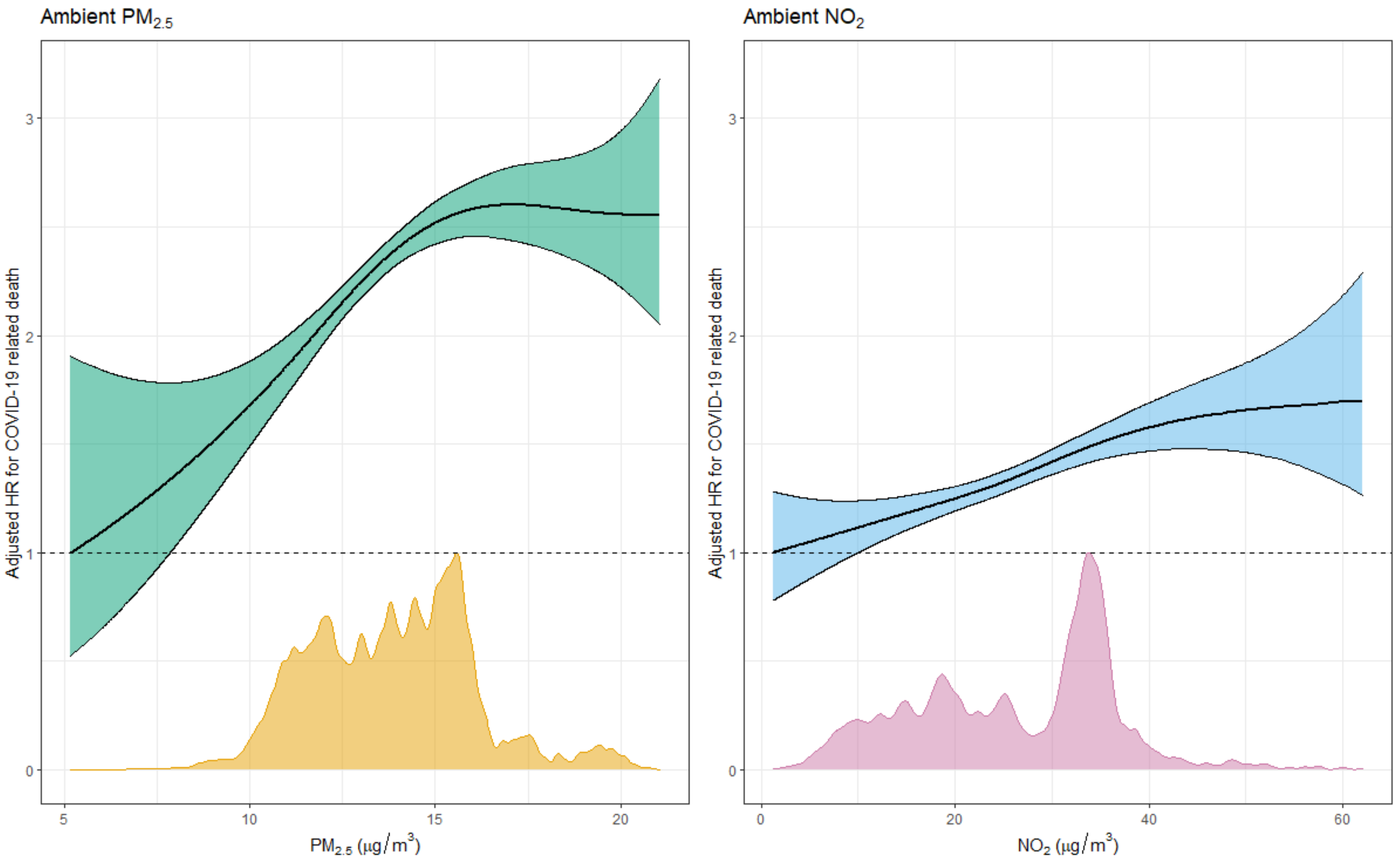

Model adjusted as Model 4: age (continuous term, penalized spline with 6 df) + sex (strata, 2 categories) + smoking status (factor, 3 categories) + individual income (factor, 3 categories) + health risk group (factor, 4 categories) + Small Area Socioeconomic Index (continuous term) + percentage of non-Spanish nationals (continuous term) + distance to the closest primary care unit (continuous term) + urbanicity (strata, 3 categories) + average weekly of test-positive proportion (continuous term) + health region (strata, 7 categories). Error bar bands refer to the 95% confidence interval from the Cox Proportional Hazards model.

Supplementary table S15. **Literature review on individual-level cohort studies and long-term exposure with severe COVID-19 outcomes**

**Population**	**Exposures**	**Outcomes**	**Reference increase**	**Findings**
**COVID-19 positive**				
1,128 COVID-19 patients diagnosed at University of Cincinnati healthcare system (UC Health)<br>(Mendy, Resp Med 2021)[9]	$PM_{2.5}$	COVID-19 hospitalization	OR per 1ug/m^3 in average and maximal $PM_{2.5}$	0.99 (0.79-1.23) for average $PM_{2.5}$<br>0.95 (0.81-1.11) for maximal $PM_{2.5}$
2,112 COVID-19 hospitalized patients in Catalonia, Spain<br>(Marquès, EI 2022)[10]	PM_{10}<br>NO_2	Severe COVID-19<br>Death	OR for categorical (High vs Low) exposure to pollutants according WHO: ≥20 for PM_{10} and ≥40 for NO_2	1.65 (1.32-2.06) for Severe COVID PM_{10}<br>2.37 (1.71-3.32) for Death PM_{10}<br><br>0.75 (0.57-0.99) for Severe COVID NO_2<br>0.77 (0.54-1.10) for Death NO_2
169K COVID-19 diagnosed US Veterans<br>(Bowe, Environ Int 2021)[6]	$PM_{2.5}$	COVID-19 hospital admission	RR per IQR (1.9 μg/m^3) annual avg $PM_{2.5}$	1.10 (1.08, 1.12)
6,524 COVID hospitalized individuals in NYC<br>(Bozack, AJRCCM 2021)[11]	$PM_{2.5}$, NO_2, BC	Mortality, ICU admission and Intubation	RR per 1 μg/m^3 annual avg $PM_{2.5}$	1.11 (1.02, 1.21) mortality<br>1.13 (1.00, 1.28) ICU<br>1.05 (0.91, 1.20) Intubation<br>No associations for NO_2 or BC
75,000 COVID diagnosed individuals in Kaiser Permanente, California<br>(Chen, AJRCCM 2022)[7]	$PM_{2.5}$, NO_2, O_3	COVID hospital admission<br>ICU admission<br>Ventilation (IRS)<br>Mortality	RR per SD (1.5 μg/m^3) in 1 yr $PM_{2.5}$	1.24 (1.16, 1.32) hospital admis (30d)<br>1.33 (1.20,1.47) IRS<br>1.32 (1.16, 1.51) ICU<br>1.14 (1.02, 1.27) Mortality (60d)<br><br>Null association with NO_2
150,000 COVID diagnosed individuals in Ontario<br>(Chen, CMAJ 2022)[8]	$PM_{2.5}$, NO_2, O_3	COVID hospital admission<br>ICU admission<br>Mortality	RR per IQR (1.7 μg/m^3) $PM_{2.5}$	1.06 (1.01, 1.12) hospital admis<br>1.09 (0.98, 1.21) ICU<br>1.00 (0.90, 1.11) Mortality

Population	Exposures	Outcomes	Reference increase	Findings
				Null association with NO_2<br>Positive association with O_3
3,139,804 individuals with COVID-19 in California (English, EA 2022)[12]	$PM_{2.5}$	Death	RR for 1 unit PM2.5<br>RR for PM quintiles	1.04 (1.03-1.05) per 1 unit<br>1.18 (1.11-1.25) for lowest quintile (9.9-11.2 ug)<br>1.56 (1.43-1.71) for highest quintile (16.2-18.8 ug)
**Non-covid cohort**				
Selected population				
9,605 individuals in Catalonia (Kogevinas, EHP 2021)[13]	$PM_{2.5}$, NO_2, BC, O_3	Severe COVID (Hospital admission/ ICU/ Oxygen)	RRR per IQR PM2.5<br>RRR per IQR NO2	1.51 (1.06, 2.16) for $PM_{2.5}$<br>1.26 (0.89, 1.79) for NO_2
424,721 individuals in England (UK-Biobank) (Sheridan, EP 2022)[14]	$PM_{2.5}$, PM_{10}, NO_2	COVID-19 hospitalization<br>COVID-19 deaths	OR per IQR PM2.5 (1.27)<br>OR per IQR PM10 (1.75)<br>OR per IQR NO2 (9.93)	1.01 (0.95-1.09) Hosp for $PM_{2.5}$<br>1.02 (0.94-1.11) Hosp for NO_2<br>1.00 (0.89-1.11) Death for $PM_{2.5}$<br>1.03 (0.90-1.16) Death for NO_2<br>Similar results for PM_{10}
General population				
1,594,308 aged 30+ residents in Rome (Nobile F, ERJ 2022)[15]	$PM_{2.5}$, NO_2	COVID-19 death	HR per IQR PM2.5 (0.92)<br>HR per IQR NO2 (9.22)	1.08 (1.03, 1.13) for $PM_{2.5}$<br>1.09 (1.02, 1.16) for NO_2

REFERENCES

1 Monterde D, Vela E, Clèries M. Los grupos de morbilidad ajustados: nuevo agrupador de morbilidad poblacional de utilidad en el ámbito de la atención primaria. Atención Primaria 2016;48:674–82. doi:10.1016/j.aprim.2016.06.003

2 Monterde D, Vela E, Clèries M, et al. Multimorbidity as a predictor of health service utilization in primary care: a registry-based study of the Catalan population. BMC Fam Pract 2020;21:39. doi:10.1186/s12875-020-01104-1

3 Meyer H, Reudenbach C, Hengl T, et al. Improving performance of spatio-temporal machine learning models using forward feature selection and target-oriented validation. Environmental Modelling & Software 2018;101:1–9. doi:10.1016/j.envsoft.2017.12.001

4 Meyer H, Pebesma E. Predicting into unknown space? Estimating the area of applicability of spatial prediction models. Methods Ecol Evol 2021;12:1620–33. doi:10.1111/2041-210X.13650

5 de Hoogh K, Chen J, Gulliver J, et al. Spatial PM2.5, NO2, O3 and BC models for Western Europe – Evaluation of spatiotemporal stability. Environment International 2018;120:81–92. doi:10.1016/j.envint.2018.07.036

6 Bowe B, Xie Y, Gibson AK, et al. Ambient fine particulate matter air pollution and the risk of hospitalization among COVID-19 positive individuals: Cohort study. Environment International 2021;154:106564. doi:10.1016/j.envint.2021.106564

7 Chen Z, Sidell MA, Huang BZ, et al. Ambient Air Pollutant Exposures and COVID-19 Severity and Mortality in a Cohort of COVID-19 Patients in Southern California. Am J Respir Crit Care Med 2022;:rccm.202108-1909OC. doi:10.1164/rccm.202108-1909OC

8 Chen C, Wang J, Kwong J, et al. Association between long-term exposure to ambient air pollution and COVID-19 severity: a prospective cohort study. CMAJ 2022;194:E693–700. doi:10.1503/cmaj.220068

9 Mendy A, Wu X, Keller JL, et al. Long-term exposure to fine particulate matter and hospitalization in COVID-19 patients. Respiratory Medicine 2021;178:106313. doi:10.1016/j.rmed.2021.106313

10 Marquès M, Correig E, Ibarretxe D, et al. Long-term exposure to PM10 above WHO guidelines exacerbates COVID-19 severity and mortality. Environment International 2022;158:106930. doi:10.1016/j.envint.2021.106930

11 Bozack A, Pierre S, DeFelice N, et al. Long-Term Air Pollution Exposure and COVID-19 Mortality: A Patient-Level Analysis from New York City. Am J Respir Crit Care Med 2022;205:651–62. doi:10.1164/rccm.202104-0845OC

12 English PB, Von Behren J, Balmes JR, et al. Association between long-term exposure to particulate air pollution with SARS-CoV-2 infections and COVID-19 deaths in California, U.S.A. Environmental Advances 2022;9:100270. doi:10.1016/j.envadv.2022.100270

13 Kogevinas M, Castaño-Vinyals G, Karachaliou M, et al. Ambient Air Pollution in Relation to SARS-CoV-2 Infection, Antibody Response, and COVID-19 Disease: A Cohort Study in Catalonia, Spain (COVICAT Study). Environ Health Perspect 2021;129:117003. doi:10.1289/EHP9726

14 Sheridan C, Klompmaker J, Cummins S, et al. Associations of air pollution with COVID-19 positivity, hospitalisations, and mortality: Observational evidence from UK Biobank. Environmental Pollution 2022;308:119686. doi:10.1016/j.envpol.2022.119686

15 Nobile F, Michelozzi P, Ancona C, et al. Air pollution, SARS-CoV-2 incidence and COVID-19 mortality in Rome – a longitudinal study. Eur Respir J 2022;:2200589. doi:10.1183/13993003.00589-2022
