# Supplementary material for: Long-term exposure to air pollution and severe COVID-19 in Catalonia: a population-based cohort study
Source: Nat Commun. 2023 May 24;14:2916. doi: 10.1038/s41467-023-38469-7 (PMC10209206; doi:10.1038/s41467-023-38469-7)
Supplement: Supplementary file 2 — Reporting Summary [file 41467_2023_38469_MOESM2_ESM.pdf]

nature portfolio

Corresponding author(s): Ranzani, OT / Tonne, C

Last updated by author(s): Apr 23, 2023

Reporting Summary

Nature Portfolio wishes to improve the reproducibility of the work that we publish. This form provides structure for consistency and transparency in reporting. For further information on Nature Portfolio policies, see our Editorial Policies and the Editorial Policy Checklist.

Statistics

For all statistical analyses, confirm that the following items are present in the figure legend, table legend, main text, or Methods section.

n/a	Confirmed	
☐	☒	The exact sample size (*n*) for each experimental group/condition, given as a discrete number and unit of measurement
☒	☐	A statement on whether measurements were taken from distinct samples or whether the same sample was measured repeatedly
☒	☐	The statistical test(s) used AND whether they are one- or two-sided<br>*Only common tests should be described solely by name; describe more complex techniques in the Methods section.*
☐	☒	A description of all covariates tested
☐	☒	A description of any assumptions or corrections, such as tests of normality and adjustment for multiple comparisons
☐	☒	A full description of the statistical parameters including central tendency (e.g. means) or other basic estimates (e.g. regression coefficient) AND variation (e.g. standard deviation) or associated estimates of uncertainty (e.g. confidence intervals)
☒	☐	For null hypothesis testing, the test statistic (e.g. *F*, *t*, *r*) with confidence intervals, effect sizes, degrees of freedom and *P* value noted<br>*Give P values as exact values whenever suitable.*
☒	☐	For Bayesian analysis, information on the choice of priors and Markov chain Monte Carlo settings
☒	☐	For hierarchical and complex designs, identification of the appropriate level for tests and full reporting of outcomes
☒	☐	Estimates of effect sizes (e.g. Cohen's *d*, Pearson's *r*), indicating how they were calculated

Our web collection on statistics for biologists contains articles on many of the points above.

Software and code

Policy information about availability of computer code

Data collection	We used R version 4.1.2 to process data.
Data analysis	We used R version 4.1.2 to perform all data analyses without any customized or new code/algorithm.

For manuscripts utilizing custom algorithms or software that are central to the research but not yet described in published literature, software must be made available to editors and reviewers. We strongly encourage code deposition in a community repository (e.g. GitHub). See the Nature Portfolio guidelines for submitting code & software for further information.

Data

Policy information about availability of data

All manuscripts must include a data availability statement. This statement should provide the following information, where applicable:

- Accession codes, unique identifiers, or web links for publicly available datasets
- A description of any restrictions on data availability
- For clinical datasets or third party data, please ensure that the statement adheres to our policy

In accordance with current European and national law, the data used in this study is only available for the researchers participating in this study. Thus, we are not allowed to distribute or make publicly available the data to other parties. Researchers can request data from the Agency for Health Quality and Assessment of Catalonia (AQuAs) by contacting by contacting the Àrea Programa d'Analítica de Dades per a la Recerca i la Innovació en Salut (PADRIS, padris@gencat.cat). Further information on the requirements and how to access the data are available at https://aquas.gencat.cat/ca/fem/intelligencia-analitica/padris/

index.html#googtrans(ca|en), including the COVID-9 prioritization procedure https://aquas.gencat.cat/ca/fem/intelligencia-analitica/padris/procediment-urgent-prioritzacio-propostes-estudis-sarscov2-covid-19/index.html#googtrans(ca|en).

Human research participants

Policy information about studies involving human research participants and Sex and Gender in Research.

Reporting on sex and gender	We used sex in this manuscript, because we have data extracted from administrative databases and sex was determined as in the birth certificate/national card identification. In this manuscript we did not provide sex-specific estimates because we aimed to evaluate the overall population-based effect of air pollution, but adjusted for sex in the models as a strata, allowing for more flexibility on capturing the effect of this covariate. Additional evaluation of sex-specific estimates are going to be evaluated in a subsequent manuscript with clear objective to assess potential effect modifiers of the association between air pollution and severe outcomes.
Population characteristics	A population-based study of all adults residing and live in Catalonia at 01/03/2020. The cohort was built with linkage of administrative databases and registries, covering the public healthcare system (nearly entire population). The average age was 54 years and 53% of females. About one quarter has hypertension and obesity, and 10% diabetes. 62% lives in urban areas, while 8% lives in rural areas, and 29% in towns/suburbs.
Recruitment	We conducted a population-based cohort of adult population, so no expected risk for selection bias.
Ethics oversight	We received approval from our local ethics committee Parc de Salut Mar Ethics Committee (CEIM-PS MAR, no. 2020/9610).

Note that full information on the approval of the study protocol must also be provided in the manuscript.

Field-specific reporting

Please select the one below that is the best fit for your research. If you are not sure, read the appropriate sections before making your selection.

☒ Life sciences ☐ Behavioural & social sciences ☐ Ecological, evolutionary & environmental sciences

For a reference copy of the document with all sections, see nature.com/documents/nr-reporting-summary-flat.pdf

Life sciences study design

All studies must disclose on these points even when the disclosure is negative.

Sample size	We did not perform a formal power analysis for this study. We aimed for a population-based cohort of whole adult population in Catalonia. Assuming the region was heavily affected during first year of the pandemic, we expected to have enough power because of the large sample size of the cohort and number of events.
Data exclusions	We described inclusion/exclusion criteria and detailed numbers excluded in the flowchart (Supplementary Figure S1). These exclusions were defined a-priori and were the basic exclusions to built the cohort, ie, to have those alive and residing in Catalonia on the start of the cohort, we excluded those who died or moved before; to ascertain a long-term air pollution level at residential address, we excluded those without a residential address geocoded and/or missing a air pollution level; to guarantee consistency in the data, we excluded those with inconsistent dates; finally, we excluded those individuals when we have lost to follow-up, ie, we dont know their vital status. All these exclusions were ≤0.1%.
Replication	We did not perform any replication experiment in this study. However, we did were several sensitivity analyses, as in epidemiological methods, using the same population, but testing different exposures, outcomes, missing data and definitions, which produced similar findings to the primary analysis.
Randomization	This is an observational study, so no randomization occurred. We used a sequential adjustment approach to reduce bias and confounding.
Blinding	This was a retrospective study, and blinding was done when possible. Thus, we designed the protocol and statistical analysis plan before extracting any data. This study did not use any kind of training, test and validation subsets.

Reporting for specific materials, systems and methods

We require information from authors about some types of materials, experimental systems and methods used in many studies. Here, indicate whether each material, system or method listed is relevant to your study. If you are not sure if a list item applies to your research, read the appropriate section before selecting a response.

Materials & experimental systems

n/a	Involved in the study
☒	☐ Antibodies
☒	☐ Eukaryotic cell lines
☒	☐ Palaeontology and archaeology
☒	☐ Animals and other organisms
☒	☐ Clinical data
☒	☐ Dual use research of concern

Methods

n/a	Involved in the study
☒	☐ ChIP-seq
☒	☐ Flow cytometry
☒	☐ MRI-based neuroimaging
